# Use of imaging technologies to support complex generic drug development

**DOI:** 10.1117/1.BIOS.3.4.043106

**Published:** 2026-10-15

**Authors:** Priyanka Ghosh, Elizabeth Bielski, Andre O’Reilly Beringhs, Bin Qin, Markham C. Luke, Sam G. Raney

**Affiliations:** U.S. Food and Drug Administration, Center for Drug Evaluation and Research, Office of Generic Drugs, Office of Research and Standards, Silver Spring, Maryland, United States

**Keywords:** complex drug products, bioequivalence, drug product characterization, imaging

## Abstract

**Significance:**

Complex drug products remain costly and inaccessible to many patients. The use of modern imaging technologies offers transformative tools to address generic drug research challenges, allowing for improved patient access to safe and effective generic medicines.

**Aim:**

This review summarizes imaging research related to a wide range of complex drug products across five coordinated initiatives: high-order characterization of the arrangement of matter in complex products; elucidation of the relationship between the structural features and the functional performance of these dosage forms; establishment of noninvasive approaches to characterize and compare the rate and extent of drug bioavailability from these dosage forms; development of reliable artificial intelligence and machine learning approaches to analyze imaging data; and provision of mechanistic insights to inform the construction of computational models.

**Results and Conclusions:**

The technologies summarized in this work enabled non-destructive structural characterization of complex drug products, establishment of structure-performance relationships, and direct measurement of drug availability at local sites of action. Furthermore, currently available technologies used for imaging complex drug products continue to support the establishment of scientifically rigorous, efficient bioequivalence pathways for complex generic products, reducing reliance on costly comparative clinical endpoint studies.

Statement of Discovery Use of imaging technologies offer transformative tools that can be used to support an evaluation of bioequivalence and expand patient access to safe, effective generic medicines.

## 1 Introduction

The Hatch-Waxman Amendments of 1984 established the Abbreviated New Drug Application (ANDA) pathway under Section 505(j) of the Federal Food, Drug, and Cosmetic Act, permitting generic manufacturers to demonstrate bioequivalence (BE) to the reference listed drug (RLD) rather than repeating clinical trials.^1^ Per the Code of Federal Regulations, BE is defined as the absence of significant difference in the rate and extent to which the active ingredient becomes available at the site of action from a generic product compared with the RLD. For small-molecule oral solid dosage forms, systemic pharmacokinetic (PK) endpoints provide a reliable and mechanistically appropriate BE surrogate. For complex drug products, however, including locally acting dosage forms, evidence based upon systemic PK endpoints is generally insufficient to support BE conclusions.^2^^,^^3^ These products are harder to genericize and remain costly (or inaccessible) to many patients; thus, as part of the FDA’s Drug Competition Action Plan (DCAP), announced in 2017, the agency has supported the development of streamlined, science-based regulatory standards for complex generic development.^4^

The Generic Drug User Fee Amendments (GDUFA), enacted in July 2012 and reauthorized in 2017 and 2022, established a generic drug research program to systematically identify knowledge gaps and corresponding research priorities to address these challenges and to improve patient access to safe, effective, and high-quality generic medicines.^5^ Through the GDUFA Science and Research Program, the FDA has supported the development of a substantial body of evidence supporting more efficient pathways by which to demonstrate BE for complex drug products. The resulting scientific insights have directly informed the FDA’s BE recommendations in Product-Specific Guidances (PSGs) and prepared the agency to provide timely, evidence-based advice to generic drug developers in pre-ANDA meetings during product development.^5^^,^^6^ The GDUFA Science and Research Program strategically coordinates two complementary strategies to reduce the reliance on comparative clinical endpoint (CCEP) BE studies: advanced product characterization methodologies and quantitative models that, together, enhance the efficiency of complex generic product development and of regulatory assessment and decision-making.^7^

Among the advanced product characterization tools developed through GDUFA-funded research, the scientific leaps in imaging technologies have arguably been among the most consequential. These advances are the result of five coordinated research initiatives to which include characterization of the arrangement of matter in complex products, elucidation of the relationship between the structural features and the functional performance of dosage forms, establishment of non-invasive approaches to characterize and compare the rate and extent of drug bioavailability, development of reliable artificial intelligence (AI) and machine learning (ML) approaches to analyze imaging data; and elucidation of mechanistic insights to inform the construction of computational models.

A key reason for the FDA’s interest in research to advance imaging technologies was to develop tools for non-destructive, *in situ*, physicochemical, and structural (Q3) characterization of the arrangement of matter within complex dosage forms. For example, for topical semisolids, X-ray microscopy (XRM), confocal Raman microscopy, and cryo-scanning electron microscopy (cryo-SEM) have enabled a three-dimensional phase resolution that was not previously achievable. For locally acting nasal suspensions, morphologically directed Raman spectroscopy (MDRS), integrating automated particle imaging with single-particle Raman chemical identification, provided the first rigorous means of characterizing the active ingredient-specific particle size distribution (PSD) in the presence of suspended excipients. For parenteral microspheres and liposomal products, focused ion beam-scanning electron microscopy (FIB-SEM) and XRM have resolved active ingredient distribution, porosity, and polymer matrix architecture at nanometer resolutions. A closely related interest was the elucidation of structure-performance correlations. GDUFA-funded research on minocycline-loaded PLGA microspheres demonstrated that FIB-SEM-derived porosity correlates positively with early in vitro drug release. It also demonstrated that XRM signal intensity variance across thousands of microspheres per batch can serve as an objective metric of intra- and inter-batch microstructural uniformity.^14^^,^^15^

Independently, imaging tools were also developed to directly measure the rate and extent to which an active ingredient becomes available at or near its site(s) of action; measuring and comparing the PK of drug delivery in situ is the essence of the regulatory concept of BE and represents the “holy grail” for assessing the BE of locally acting products. In the skin, advances with confocal Raman spectroscopy and stimulated Raman scattering (SRS) microscopy facilitated the acquisition of spatially and temporally resolved drug concentration data. Similarly, in the eye, advances with matrix-assisted laser desorption/ionization mass spectrometry imaging (MALDI-MSI) and ocular fluorophotometry enabled biodistribution mapping and quantification of clearance kinetics.

In parallel, AI/ML analytical methods were developed as an essential tool to analyze complex multidimensional imaging datasets. Supervised ML algorithms such as U-Net convolutional neural networks, among others, were used to identify lipid-containing regions as lipid-rich and the surrounding regions as lipid-poor in the skin, for example, in an effort to apply validated feature recognition uniformly across massive datasets, converting raw structural data into suitable quantitative metrics.

Finally, the mechanistic insights generated through each of these research initiatives were leveraged to inform the development of mechanistic computational models. Notably, this included Digital Image-Based Modeling (DIGI-M) frameworks that were able to simulate how microstructural differences would translate to differences in drug release and bioavailability.

Along with remarkable innovations in imaging technologies and analytical methods during the last decade, the FDA’s GDUFA research initiatives have helped to establish new approaches by which to support demonstrations of BE for complex drug products. These new approaches may finally make it possible to assess BE for many complex products in the manner that the HatchWaxman Amendments conceived, and they certainly offer powerful ways to facilitate the pharmaceutical development and regulatory assessment of some of the most challenging complex products.

## 2 Advances in Imaging Technologies for Characterization of Arrangement of Matter

### 2.1 Characterization of Products Applied to the Skin

Products applied to the skin are available in a wide variety of dosage forms, ranging from simple solutions to semisolid gels, ointments, lotions, and creams that are typically available in an aluminum tube or a plastic bottle as the container closure system (CCS), resulting in a wide range of arrangements of matter for the products dispensed on the skin for drug delivery. An additional level of complexity arises for products that are solutions or emulsions *in situ* but change form and microstructure when dispensed, such as foams or sprays. And finally, there is a wide range of products designed as delivery systems, ranging from swabs to adhesive-based systems that are analogous to bandages but contain drugs. Historically, establishing BE for this group of products predominantly relied on resource- and time-intensive CCEP BE studies because there was a lack of understanding of the structure-function relationship for these products. More recently, GDUFA-funded research has led to the development of “totality-of-evidence” based *in vitro* characterization approaches for establishing BE of these products, which include extensive drug product characterization including those using optical microscopy (bright field or polarized) and light diffraction techniques (X-ray diffraction and XRD) for assessment of PSD and globule size distribution (GSD) as well as performance tests such as comparative evaluation of release and permeation.^8^ Some examples of such PSGs include those for acyclovir topical cream, diclofenac sodium topical gel, tacrolimus topical ointment, etc.,^9^^–^^11^ However, historically, advanced imaging technologies have not been used for high-order characterization of the arrangement of matter, such as distribution of the active ingredient in the formulation or categorization of the phases in the formulation, both of which in turn may influence the performance of these dosage forms. This may be in part because most of the topically applied formulations tend to contain a large amount of water or another solvent system that undergoes rapid evaporation and therefore it is challenging to stabilize the product in its original form for image-based characterization.

Below we outline some recent examples of how advanced imaging tools have been used to generate high-order characterization data for such products.

Yeoh et al.^12^ reported the use of X-ray microscopy (XRM) for 3D characterization of multiphasic semisolid formulations, including a petrolatum-based ointment and an oil-in-water emulsion based cream. The sample preparation involved transferring a very small volume of the sample (5  μl) into a microcentrifuge tube, followed by scanning the samples using a Zeiss Versa 520 XRM instrument. 4000 to 5000 tomographic projections were acquired for each of the cream and ointment samples over 9 h, the projections were subsequently used to generate a 3D digital presentation of each sample. The authors used additional processing tools to quantify the relative distribution of phases (e.g., oil versus water) in the samples as well as AI-based image segmentation to further inform the microstructure. The authors concluded that XRM in combination with the image analysis tools employed in the study allows us to have a comprehensive understanding of the microstructure of a topical semisolid product beyond PSD and GSD. However, use of the technique for topical semisolid drug product characterization and comparison of formulations during product development is tricky because it takes relatively long to collect the data, and one needs to be careful not to overheat the formulation, which in turn could lead to changes in the microstructure of these semisolids.

Fujii et al.^13^ reported the use of attenuated total reflection Fourier transform infrared (ATR FT-IR) spectroscopy for characterization and comparison of the distribution of different components in seven tacrolimus topical ointments. The methodology was able to illustrate the difference in distribution of the components in the ointments, for example, that polycarbonate tends to form liquid droplets that disperse in the petrolatum in these formulations. One limitation of the technique, however, was that detection was limited to the surface of the formulation. The authors, therefore, also attempted to look across the depth of the formulations using confocal Raman spectroscopy to more accurately quantify both the microstructure and the relative distribution of components in the formulation overall.

Mohammed et al.^14^ reported the use of optical microscopy, confocal Raman microscopy, and cryo-SEM to evaluate the microstructure of acyclovir topical creams that are dispensed using different CCS (a tube or a pump). Although it is unknown if there were any differences in components and composition between the products that were inside the two CCS, the study revealed that dispensing the product using a pump compared to the tube impacted the microstructure. Specifically, the optical microscopy data confirmed that globules were only visible in the samples dispensed from the pump. The confocal Raman data generated using a confocal Raman microscope (alpha300 R+, WITec GmbH, Ulm, Germany) concluded that the globules consisted of dimethicone and were potentially generated due to the mechanical stress experienced by the product when extruded through the nozzle of the pump.

Topical formulations applied to the skin are generally complex, containing anywhere between 4 and 15 components to be both pharmaceutically efficacious and cosmetically elegant. The three recent examples from the literature illustrate how researchers are currently using a wide array of both microscopy and spectroscopy tools, or a combination thereof, to enhance our understanding of the arrangement of matter in topical semisolid products. With additional research in this area, such data can be used to establish structure-performance correlations and support an assessment of BE.

### 2.2 Characterization of Products Applied to the Eye

The efficient development of complex generic ophthalmic products relies on the demonstration that the proposed generic product is not only qualitatively (Q1) and quantitatively (Q2) the same as the RLD but also possesses the same Q3 characteristics. Because the eye possesses highly efficient clearance mechanisms, such as rapid tear turnover and nasolacrimal drainage, minor differences in formulation microstructure can significantly alter a drug’s ocular residence time, dissolution rate, and ultimately, its bioavailability.^15^^–^^18^ Consequently, “totality-of-evidence” approaches to establish BE for these products are generally desired, and these rely heavily on advanced *in vitro* and *in vivo* imaging techniques to support the conclusion that the generic product and RLD are physically, structurally, and functionally the same.

Ophthalmic suspensions are complex multiphase systems where the active ingredient is dispersed as solid particles within an aqueous vehicle. The PSD and morphology of the active ingredient are critical quality attributes (CQAs) because they directly govern the drug dissolution rate in the limited volume of tear fluid.^19^^,^^20^ FDA-supported studies with indomethacin, budesonide, and brinzolamide suspensions show that PSD and viscosity jointly influence suspension stability, dissolution/release, and ocular absorption; together, these findings justify treating PSD as BE-relevant CQA rather than merely routine quality descriptors.^16^^,^^18^^,^^19^ However, traditional bulk particle sizing methods, such as laser diffraction, cannot distinguish between the active ingredient and insoluble excipients or agglomerates, limiting their utility in comparative evaluations of generic products. To overcome this limitation, Morphologically Directed Raman Spectroscopy (MDRS) has emerged as a powerful imaging tool for establishing Q3 sameness in complex suspensions. MDRS integrates automated static imaging with Raman spectroscopy, allowing for the isolation and chemical identification of individual particles within a formulation. By applying MDRS, developers can generate active ingredient-specific PSDs and morphological profiles (e.g., circularity and elongation) without interference from suspended excipients. Although ophthalmic-specific MDRS publications remain limited, FDA-funded MDRS work in locally acting nasal suspensions is analytically relevant here because it demonstrates ingredient-specific particle sizing in complex suspensions where conventional bulk methods cannot distinguish active ingredient from excipient particles (See Sec. 2.3). This technique is particularly valuable for products such as loteprednol etabonate or tobramycin/dexamethasone suspensions, where demonstrating that the generic active ingredient particles mirror the size and shape of the RLD is a pivotal component of the *in vitro* BE weight-of-evidence.^21^^,^^22^

Ophthalmic emulsions (e.g., cyclosporine ophthalmic emulsion) present unique characterization challenges due to their thermodynamically unstable nature. The size and distribution of the dispersed oil globules are critical for ensuring consistent drug delivery, physical stability, and ocular tolerability. Although dynamic light scattering (DLS) is commonly used for sizing, it provides an ensemble average and may obscure the presence of larger, outlier globules that indicate physical instability or differ from the RLD. For cyclosporine ophthalmic emulsions, FDA research has emphasized orthogonal droplet size characterization rather than reliance on a single sizing method. For these products, asymmetric flow field-flow fractionation can resolve polydisperse globule-size distributions more sensitively than batch-mode dynamic light scattering (DLS) alone, and later integrate cryo-TEM, nanoparticle tracking analysis, diffusion-ordered NMR, and biphasic drug-transfer models into a quality-by-design framework for comparative product assessment.^23^^,^^24^ This body of work helped support review and approval of the first US generic 0.05% cyclosporine ophthalmic emulsion.

Furthermore, for semi-solid ophthalmic ointments, FDA-funded research has clearly shown that microstructural sameness is reflected in rheology and release behavior. Q1/Q2-equivalent ointments prepared with different excipient sources or processing routes can exhibit different rheological properties and release profiles despite similar composition.^25^^–^^29^ This reflects the importance of imaging-based microstructural characterization as supportive evidence of product performance sameness in terms of its potential impact on drug release.

A growing area of complex generic development centers on ophthalmic long-acting implantable and insertable products designed for extended, controlled drug release into the posterior or anterior segments of the eye (e.g., dexamethasone and fluocinolone acetonide implants). These products rely on biodegradable or nonbiodegradable matrices to govern the slow dissolution and release of the active ingredient over several months. Because conducting traditional PK studies for long-acting ophthalmic implants is practically and ethically challenging, and due to limitations around CCEP BE studies for ocular indications, rigorous *in vitro* structural characterization together with *in vitro* release testing is central to equivalence assessment. It is also reflected in current ophthalmic PSGs; for example, FDA’s 2025 draft PSG for dexamethasone intravitreal implant recommends a characterization-based pathway centered on a totality of evidence *in vitro*-only approach, where advanced imaging is crucial for comparatively characterizing the internal and external architecture of these solid dosage forms.^30^ X-ray Computed Tomography (X-ray CT) and XRM are employed to nondestructively visualize the internal 3D structure of the implant, precisely quantifying total porosity, pore size distribution, and the presence of internal voids or manufacturing defects.^31^^,^^32^ Differences in internal porosity between a generic implant and the RLD can alter hydration rates and subsequent drug release profiles. Recent FDA research on dexamethasone-loaded ophthalmic implants and inserts further showed that excipient-source variability can propagate into hydrogel porosity, pore architecture, mechanical strength, swelling, crystallinity, and release rate, and that XRM coupled with AI-based image analysis can sensitively resolve these structure–performance relationships. Demonstrating that a generic implant possesses an identical active ingredient-polymer dispersion and structural characteristics to the RLD is essential for ensuring that the mechanism of controlled release remains fundamentally the same.

Qualitative imaging analysis generally requires imaging segmentation of material phases. Large imaging datasets are very common. As it relates to imaging data for product characterization, ∼500 to 1000 2D cross-section images (20 to 30 nm slice thickness) may be needed to construct a 3D volume of one microsphere. For statistical representativeness, often thousands of microspheres per batch are analyzed in a single XRM scan. Therefore, manual processing of such large-scale imaging data is impractical. In addition, conventional image processing algorithms may struggle with challenges such as similar grayscale histograms, varying contrast, and artifacts that obscure small features. Supervised AI offers a solution by identifying patterns that traditional methods miss, enabling validated features to be automatically applied across massive datasets. For Q3 data, deep learning algorithms, particularly convolutional neural networks, are increasingly utilized to automate image segmentation and particle classification. For example, in the evaluation of complex ophthalmic suspensions, AI models can be trained on MDRS or related pharmaceutical particle-imaging datasets to automatically distinguish active ingredient crystals from similarly sized excipient agglomerates based on subtle morphological and spectral signatures. This ensures that the evidence of microstructural sameness is objective and reliable. Another example of AI-enabled image analysis includes FDA work on dexamethasone-loaded ophthalmic inserts, which used AI-based image analysis of X-ray microscopy data to quantify microstructural differences that correlated with swelling and release behavior.^31^

Overall, FDA’s ophthalmic regulatory science program has already produced concrete translational outcomes. A recent success includes FDA research on cyclosporine ophthalmic emulsion, which established and set expectations for orthogonal characterization methods that helped support approval of the first US generic for cyclosporine ophthalmic emulsion 0.05%. More broadly, FDA’s recent ophthalmic quality guidance and GDUFA priorities reinforce a characterization-based BE paradigm for complex ophthalmics, including long-acting inserts/implants, complex formulations, combination products, and AI-enabled assessment tools.^33^

### 2.3 Characterization of Locally Acting Nasal and Inhalation Drug Products

Imaging technologies have been extremely critical for BE evaluation of complex, locally acting, generic nasal and inhalation drug products. Understanding the plume behavior of the emitted dose from the device, as well as drug and droplet particle sizes, morphology, and/or agglomeration behavior of nasal and inhalation formulations, provides evidence that can support the understanding of how such drug products become available at the local site of action. Such imaging is generally recommended for these products when they contain more complex formulations, e.g., suspension-based and powder formulations that require the active ingredient to be dissolved upon reaching the target site. With the advancement of sensitive imaging technologies and the support of GDUFA research initiatives, updates to the PSGs of nasal and inhalation drug products to replace less sensitive and more burdensome CCEP BE studies have been possible. These imaging techniques are reflected in the most recent versions of the PSG, including recommendations such as *in vitro* BE or comparative characterization studies.

For locally acting nasal suspensions, CCEP BE was initially recommended as part of a “weight-of-evidence” approach for which a totality of evidence was recommended to ensure BE, including formulation sameness, device similarity, in vitro studies to ensure equivalent product performance, and PK and CCEP BE studies to ensure equivalent systemic drug exposure and clinical effect.^34^ Specifically, for suspension nasal drug products, the understanding of the drug particle size is considered a key factor influencing local bioavailability within the nose, as it impacts local deposition, dissolution, and rate of mucociliary clearance. However, the inability to adequately measure drug particle size within a formulation that also contained suspended excipients was a critical reason additional *in vivo* studies were recommended to ensure BE of a locally acting generic suspension-based nasal drug product.^35^ Such challenges were addressed by the advent of MDRS, an imaging technique capable of chemical identification and imaging. As noted in the previous section, MDRS uses Raman spectroscopy to chemically identify the particles in the sample and selection of particles exhibiting specific morphological features via morphology filters that allow one to conduct particle size characterization on the selected population of interest.^33^ This has been demonstrated by successful drug particle size characterization of mometasone furoate within mometasone furoate nasal spray suspensions in the presence of suspended microcrystalline cellulose and carboxymethylcellulose sodium excipient particles with MDRS.^33^^,^^36^^–^^38^ Such characterization also provided data to support the idea that drug particle size can impact both dissolution and systemic absorption of the drug, further strengthening its importance in influencing local and systemic bioavailability.^36^^,^^37^ Thus, MDRS has allowed for characterization of drug particle size in the presence of suspended excipients accurately within a nasal suspension drug product and supported the approval for the first generic mometasone furoate metered nasal spray.^33^ This has allowed the FDA to recommend in vitro characterization of drug particle size distribution using MDRS or similar techniques as one of the in vitro BE studies in PSGs for suspension-based metered nasal spray drug products in lieu of conducting the less sensitive and costly CCEP BE studies.^39^ However, the greatest limitation for MDRS currently is its inability to measure particle sizes with <1  μm.^33^^,^^35^ Thus, new imaging techniques capable of measuring particle sizes <1  μm and carry the same level of sensitivity in chemical identification are an area of further exploration.

Although emphasis on imaging analysis for locally acting suspension-based nasal spray drug products has been on drug particle size characterization, for locally acting inhalation drug products, such as metered-dose inhalers (MDIs) and dry powder inhalers (DPIs), active ingredient PSD is already well characterized by their aerodynamic particle size distribution (APSD), typically measured by cascade impaction. Along with APSD, particle morphology (e.g., shape, surface roughness, porosity, and crystalline/amorphous structure) of the residual aerosolized particles, once deposited in the lung, can impact the rate of dissolution of the active ingredient and cellular permeability and uptake within the lung (BA at the site of action).^40^^–^^42^ Therefore, morphology imaging comparisons of the aerosolized dose are recommended as a characterization study in the PSGs for locally acting MDIs and DPIs to compare the residual particle morphology and agglomeration behavior for those cases with complex inhalation formulations that have unique excipient particle technologies or engineered particle formulations that influence product performance, have multiple drug components, or are dry powder formulations for which manufacturing, device, and formulation play critical roles on the drug deposition state. Such data can support the understanding of how particle morphology characteristics impact the BA of the drug once deposited within the lung, as well as understanding any potential formulation differences between generic and RLD inhalation drug products. To support these studies, GDUFA research has included exploration of imaging techniques for locally acting inhalation drug products that have spanned a range of various imaging modalities depending on the unique aspects of the inhalation formulation, some of which are highlighted here.

The FDA is currently exploring the application of optical photothermal infrared (O-PTIR) spectroscopy and microscopy to characterize the drug, drug-drug, and drug-excipient agglomerates present in the aerosolized fraction from a fluticasone propionate (FP); salmeterol xinafoate (SX) DPIs via Contract 75F40122C00202 with the University of Sydney and the University of Alabama at Birmingham. O-PTIR provides a sensitive method for assessing the chemical-specific identification of components and agglomerate behavior of the emitted dose at the ingredient-specific and micron and submicron scale for inhalation powders, an improvement on MDRS, which is limited to the micron scale (resolution of 1  μm). O-PTIR instruments generally have ≤0.5  μm x-y resolution with the sensitivity to detect signal from particles as small as ∼0.2  μm.^43^ IR spectra can be measured from single particles, particle subdomains, and map out particle and particle agglomerate locations, chemically identifying each component within regions of interest that can span up to hundreds of microns in size, demonstrating its versatility in analyzing single particles as well as large particle populations of interest. Raman spectra can also be captured simultaneously with the IR measurement, as Raman scatter is also produced by the probe laser within the O-PTIR instrument. By employing O-PTIR imaging on aerosolized fractions of FP; SX DPIs, drug-drug and drug-excipients were successfully identified, characterized by their particle size, morphology characteristics, and composition. Statistical analyses revealed significant differences between drug or excipient components, drug products, and aerosolized fractions, demonstrating great sensitivity.^44^^,^^45^ Current work is ongoing to relate these outcomes to other common *in vitro* performance metrics measured for inhalation powders, such as the APSD of FP and SX and the dissolution behavior of FP.

Work within Contract 75F40122C00202 is also exploring atomic force microscopy-infrared spectroscopy (AFM-IR) to characterize FP and SX DPIs. AFM-IR has the advantage of combining the spatial resolution of AFM below the typical optical diffraction limits, as well as providing chemical identification via the IR spectroscopy.^46^ In this work, AFM-IR was able to successfully generate bright field images, direct current images, and single wavenumber chemical images for FP, SX, and lactose and agglomerates from an FP; SX DPI.^47^ Thus, high-resolution images and chemical composition of particle agglomerates in the submicron range were successfully characterized. A main benefit of this technique is the high-resolution images provided that can provide great characterization of the morphology of small agglomerates. However, upscaling the technique to provide image analysis on a larger particle population scale was seen as a great challenge, for which O-PTIR is better suited.

A recent focus for MDIs has been the transition of MDIs from hydrofluoroalkane (HFA) propellants to next-generation propellants (NGPs).^48^ Thus, several imaging modalities within Contract 75F40123C00186 with Aptar Pharma have also been explored to analyze propellant properties, gasket valve material properties, and compare drug morphology changes from the emitted dose for MDIs that differ in propellants. To observe the propellant evaporation process of HFA 134a, HFA 152a, and HFO 1234ze at various relative humidities (50% and 90% RHs), a modified stroboscopic droplet analysis (SDA) technique coupled with the electrodynamic balance (EDB) method was employed.^49^ The SDA analysis comprised of a stream of uniform droplets is generated from the propellants by a droplet-on-demand dispenser and imaged using stroboscopic imaging. The EDB allowed the droplets generated from the droplet-on-demand dispenser to be charged and trapped in an electrodynamic field for which light scattering is collected and analyzed to calculate droplet size. The SDA coupled with EDB demonstrated that the evaporation rate of HFO 1234ze was ∼4 times faster as compared to HFA134a and around twice that of HFA152a. In addition, water condensation, when compared with 50% RH to 90% RH, produced negligible changes to the evaporation dynamics of these propellants.

Lastly, SEM was also used to evaluate the drug particle morphology of the emitted dose of beclomethasone dipropionate MDIs and albuterol sulfate MDIs, which were formulated using various propellants: HFA-134a, HFA-152a, and HFO-1234ze.^50^ Both beclomethasone dipropionate MDIs and albuterol sulfate MDIs did not demonstrate any significant changes in particle morphology from the emitted dose, suggesting that the propellants had little impact on influencing drug particle morphology. Such data helps support changes between propellants for these MDIs such as providing data that allows for assessment of critical aspects that may potentially impact drug product performance as well as drug deposition and absorption. Overall, imaging modalities have played a critical role in multiple aspects, form excipient characterization to product performance of MDIs when considering changing the main excipient, the propellant system. These techniques help support quality and BE aspects of generic product and brand-name product development and help to reveal the critical attributes to consider for the NGP transition.

Unique inhaled formulations with engineered particles have also benefited from imaging assessment. For example, assessment of spray-dried phospholipid porous particles (PPPs) included them as excipient particles as a drug carrier and co-suspending agent in MDIs and DPIs. Such PPPs were designed to provide physical stability in suspension-based MDIs, provide consistent dosing across the unit life, ease aerosolization dispersibility, and improve aerodynamic properties.^51^^–^^53^ As such, a study supported by an FDA Office of Chief Scientist CORES Grant, including imaging assessment, was conducted to analyze the particle surface morphology of PPPs from TOBI PODIHALER (tobramycin inhalation powder), BEVESPI AEROSPHERE (formoterol fumarate; glycopyrrolate metered inhalation aerosol), and BREZTRI AEROSPERE (budesonide; formoterol fumarate; glycopyrrolate metered inhalation aerosol) using SEM. SEM images were subsequently analyzed by artificial intelligence-machine learning (AI-ML) segmentation using DigiM I2S to extract particle size distribution (PSD), surface pore size distribution, and surface roughness of the PPP emitted from these products.^54^ The AI-ML analysis of the SEM images successfully characterized PSDs similar to those measured by other techniques (APSD by cascade impaction and laser diffraction) as well as well providing morphological assessments. Higher surface roughness was identified for PPPs from BREZTRI AEROSPERE, which also demonstrated a higher fine particle fraction (FPF) and smaller median mass aerodynamic diameter (MMAD), indicating more efficient aerosolization as compared with BEVESPI AEROSPHERE, despite the PPPs PSDs being similar. Therefore, it was suggested that the surface roughness and morphology appear to be significant factors likely reducing interparticle drug cohesion and easing the drug aerosol dispersion. Such work has begun to help identify key drug product characteristics of these PPPs that appear to influence aerosolization behavior. AI-ML analysis on images generated by 2D Focused Ion Beam Scanning Electron Microscopy (FIB-SEM) has also been used to further explore the internal structures of PPPs emitted from TOBI PODIHALER to expand analysis of the porous structures beyond the surface. The use of SEM, FIB-SEM, and AI-ML analysis is likely to support similar work in the future when complex formulations with engineered particles are employed in MDIs and DPIs.

### 2.4 Characterization of Parenteral Microspheres and Liposomal Drug Products

Imaging technologies are increasingly employed to support both product development and BE determination for generic parenteral complex formulations. While systemic PK studies remain the “standard” approach for establishing BE in many of these complex drug products, a “totality of evidence” approach becomes necessary when systemic PK data alone are insufficient to ensure BE or when an *in vitro* characterization-based BE approach is recommended. In such cases, imaging technologies provide critical information on the structural arrangement of constituent materials, which serves as meaningful supportive evidence in the overall BE assessment.

For example, the PSGs for liposomal products consistently recommend demonstrating sameness in liposome morphology, number of lamellae, and the state of the encapsulated drug, in addition to an *in vivo* PK BE study.^55^^,^^56^ In another example, the PSG for minocycline hydrochloride extended-release microspheres recommends an *in vitro*-only BE approach, in which microsphere microstructure is included as part of the comparative characterization studies to support BE determination.^57^ Advanced imaging techniques, such as Focused Ion Beam-Scanning Electron Microscopy (FIB-SEM), XRM, and Cryo- Electron Microscopy (Cryo-EM) have been used to characterize the microstructure of complex parenteral drug products. These techniques enable visualization and quantification of Q3 attributes that impact drug release and performance.

Cryo-EM has been widely used as a powerful tool for elucidating biomolecular structure. Unlike conventional EM conducted at room temperature, cryo-EM “freezes” molecules in their native hydrated state, enabling visualization of biomolecular structures without the artifacts introduced by traditional sample preparation methods such as dilution and dehydration. The preservation of native structures makes cryo-EM very valuable for characterizing parenteral injectable products with complex internal architectures and supplied in aqueous vehicles, such as liposomes,^58^ emulsions,^59^ and iron complexes.^60^

EXPAREL (bupivacaine multivesicular liposome), developed using DepoFoam technology, possesses a unique honeycomb-like multivesicular structure. The PSG specifies that a generic product should demonstrate comparable structure and morphology, including the internal honeycomb architecture, to the RLD. Since this internal structure is fragile and susceptible to collapse during conventional sample preparation, cryo-SEM provides a minimally disruptive approach that preserves the native architecture, allowing characterization of the honeycomb architecture.^61^

Propofol is formulated as an oil-in-water intravenous emulsion stabilized by egg lecithin. Because excess lecithin phospholipids can form a variety of non-droplet structures, and the relationship between these structures and drug product performance remained poorly understood, cryo-TEM was employed to characterize four therapeutically equivalent propofol products. Cryo-TEM enables accurate visualization of structural components without the artifacts introduced by conventional drying techniques. Cryo-TEM analysis revealed that all four products contain oil droplets, lipid vesicles, and oil droplet–lipid aggregates; however, the size and abundance of lipid vesicles and oil droplet–lipid aggregates varied across products. Despite these structural differences, all four products exhibited comparable GSD as measured by dynamic light scattering and laser diffraction. Because propofol was found to localize predominantly within the oil droplets, the observed differences in lipid vesicles and oil droplet–lipid aggregates are not expected to meaningfully affect drug product performance.^58^

Intravenous iron complex products consist of an iron oxyhydroxide core surrounded with a carbohydrate shell, used to treat iron deficiency. The particle size of the iron core can impact iron lability and, consequently, the safety and efficacy of these products. For generic iron complex products, the FDA considers the particle size and morphology of the iron core as key parameters in demonstrating sameness to the RLD. A survey of the literature reveals inconsistent results in iron core particle size determination, even when the same analytical techniques are employed. These reports typically relied on TEM conducted at room temperature. A study was conducted to characterize four iron complex products using Cryo-TEM compared to conventional room-temperature TEM. The results demonstrated that sample preparation at room temperature causes particle aggregation and deformation. These artifacts are believed to contribute to the inconsistent results observed in the literature. In contrast, Cryo-TEM preserves the products in their native, frozen-hydrated, and undiluted state, enabling visualization of the ultrafine structure of the iron cores.^59^

For polymeric microspheres and implant products, understanding microstructure can provide insight into drug release mechanisms, support the development of predictive modeling tools, and help establish sameness. Due to the size of these dosage forms, TEM is not applicable for visualizing internal structure. Instead, slicing or cross-sectioning of the sample is required. FIB-SEM is a microstructural characterization technique that has been extensively employed in materials science and the semiconductor industry and has more recently emerged as a promising tool for the characterization of pharmaceutical samples. Using a proper focused ion beam (usually gallium) to hit the specimen surface, a small amount of material on the sample can be precisely removed, and the inner structure can be imaged by SEM.^62^ Through repeated milling and imaging, the 3D topography of the sample can be reconstructed. FIB milling offers advantages over conventional mechanical cutting techniques by minimizing certain artifacts. A comparative study using minocycline hydrochloride microspheres demonstrated that conventional mechanical cutting introduces residual surface topology from fracturing, whereas FIB milling produces clean cross-sections that clearly reveal distinct material phases.^63^ The internal structure of a microsphere or implant typically consists of three material phases: active ingredient, polymer, and pores. Depending on active ingredient properties and manufacturing process, the active ingredient within microspheres may exist either as a distinct phase or dispersed molecularly within the polymer phase.^64^ In-SEM Energy-dispersive X-ray spectrometer^63^ or Raman spectroscopy^65^ mapping can be used to confirm these different material phases by detecting elemental differences between the active ingredient and polymer.

Quantitative image-based microstructure analysis from FIB-SEM includes size and spatial distribution of active ingredient particles and pores, etc. Using minocycline hydrochloride microspheres as a model drug, the correlation between manufacturing process variables, microstructure, and drug release was investigated across eight PLGA formulations prepared under varying processing conditions (stirring method and speed, coacervation parameters), along with the commercial product Arestin.^66^ 2D FIB-SEM cross-sectional images revealed clear phase separation between active ingredient and polymer, with consistent active ingredient D50 value across all nine formulations, indicating that the coacervation process does not change active ingredient particle size. In contrast, pore D50 varied significantly with manufacturing conditions, and a positive correlation was observed between porosity and Day 1 *in vitro* drug release. Five of the nine samples (eight formulations and the commercial product), were further characterized by 3D FIB-SEM. The 3D active ingredient particle size distribution was similar to that determined from 2D images. The porosity and pore size determined from 3D FIB-SEM followed the same rank order as the 2D analysis, though 3D data showed higher porosity values due to the inherent limitation of 2D cross-sections in capturing the full pore network. The positive correlation between 3D porosity and Day 1 drug release was confirmed, though the slope was smaller than that observed with 2D data. The spatial distribution of active ingredient and pores within microspheres was linked to *in vitro* drug release from the following perspectives: (1) Multiphasic release profiles were consistently associated with nonuniformity in either the active ingredient or pore spatial distribution, or both. In contrast, the sample with uniform active ingredient and pore distribution (sample D1397) produced continuous, lag-free release, because the active ingredient and pore networks were well-connected throughout the sphere, enabling consistent diffusion pathways from surface to core. (2) Sample with nonuniform active ingredient distribution (D1370, where the active ingredient was depleted near the surface) had a reduced initial burst release, followed by lag phase, because the outer shell lacked accessible drug. (3) Sample with nonuniform pore distribution showed slower initial release, as the pre-existing pore network near the surface was limited, delaying media ingress and drug diffusion.

Despite its high resolution for revealing microstructure, one limitation of FIB-SEM is that this method characterizes one microsphere at a time (∼12  h each), making large-scale characterization impractical. Complementary methods are needed to confirm that the selected microspheres are representative of the test sample. XRM may serve this purpose, as it is capable of imaging thousands of microspheres simultaneously at submicron resolution. X-ray signal intensity is dependent on elemental composition and spatial differences in density. For compositionally identical samples (e.g., generic product versus RLD), variations in XRM signal intensity will reflect differences in microstructure—specifically, porosity and active ingredient loading.

XRM can be used to inform the intra-batch structural uniformity of polymer microspheres. Clark et al. characterized eight batches of minocycline hydrochloride microspheres that differ in process parameters. For all eight samples, the X-ray signal intensity is very similar for microspheres greater than 20  μm, suggesting great intra-batch structural uniformity. The relatively higher variability observed in small-sized microspheres (<10  μm) is because these microspheres are more susceptible to intensity variations due to individual drug particles, large pores, and lower signal-to-noise ratios at limited image resolutions. Batch-to-batch (or test versus RLD) comparisons cannot be directly assessed using X-ray signal intensity alone. Instead, the variance in signal intensity per microsphere should be used as the primary metric. A high variance would suggest a non-uniform distribution of phases within the microspheres, whereas a low variance would indicate greater homogeneity.^67^ In addition, microsphere density measurements can be performed when calibrated with standards that were imaged concurrently with the samples. The density measurement can detect changes in microsphere density resulting from polymer aging, and these changes were found to be consistent with the porosity changes measured by FIB-SEM.^68^

## 3 Advances in Imaging Technologies for Characterization of Rate and Extent of Drug Delivery

### 3.1 Drug Delivery to the Skin

Skin is the largest and most accessible organ of the human body, and yet historically it has been challenging to quantify the amount of drug delivered into and across the skin. Therefore, drug development for products applied to the skin has largely relied on large comparative clinical endpoint studies in hundreds to thousands of subjects for establishing efficacy. This has been the gold standard for both new “brand” name drugs as well as follow on generic drugs in the US and across the world. However, as mentioned in the introduction, within the scope of the GDUFA science and research program the FDA has prioritized regulatory science research to evaluate alternative methodologies, including a wide array of imaging tools, to facilitate evaluation of drug delivery at or near the site of action.

In one of the earliest examples of using label-free chemical imaging for quantification of drug distribution in the skin, Saar et al.^69^ reported the use of a LabRam HR, Horiba Jobin Yvon Raman microspectrometer, for quantification of ibuprofen and ketoprofen. The two molecules were applied to mouse skin as solutions in propylene glycol, which was deuterated, and permeation of the two molecules was quantified in the skin across different depths and over time. The work represents one of the first illustrations of how nondestructive imaging tools can be used to elucidate the formulation metamorphosis and permeation of active and inactive ingredients in the skin from topically applied formulations. The authors reported that one of the major challenges was analyzing the 3-D data to extract quantitative information, which is critical for comparing formulations. The other potential limitation was that some of the molecules had to be deuterated for visualization against the background of the skin tissue.

Skin being so readily accessible (as compared to the tissues in the lungs, for example) is an obvious target for direct assessment of drug delivery from drug products using imaging tools. Within the scope of the research program, FDA-funded work has evaluated the use of spectroscopy-based tools for label-free noninvasive measurement of drug concentration in the skin.

Jung et al.^70^ reported the use of an Alpha300R+ confocal Raman microscope from WITec (WITec GmbH, Ulm, Germany) for quantification of acyclovir in the skin from six different acyclovir cream formulations at two different time points at 4 h and 24 h following topical application of the creams. The creams were known to have differences in formulation, and the data illustrated that the cumulative amount of acyclovir in the skin was significantly higher from the US RLD compared with the “generic” formulations utilized in the paper, illustrating the ability of the confocal Raman-based tool to evaluate and compare drug delivery from topical formulations in the skin at different timepoints. Other available datasets from IVPT studies^71^ as well as *in vivo* dOFM studies^72^ corroborate the findings from the confocal Raman study, all of which consistently illustrate that the bioavailability of acyclovir from the US RLD is higher compared to the nonUS approved “generic” formulation(s) used in the papers. One potential limitation of the dataset may be that the skin concentrations using confocal Raman were measured at only two timepoints, which may not be sufficient to assess both the absorption and the elimination of acyclovir from the skin as would be expected in a typical PK study.

Zarmpi et al.^73^^,^^74^ used confocal Raman spectroscopy (Renishaw RM1000 Raman microscope) to compare the bioavailability of multiple metronidazole and doxepin formulations over 16 h following topical application to the skin *ex vivo*. The studies were designed such that the reference formulation was compared with itself to ascertain the variability associated with the measurements themselves. In addition, the reference formulation was compared to an approved US generic product, which was expected to be BE (had been previously determined to be BE compared with the RLD using standardized BE approaches), and alternate lab-made formulations, which were designed to be bioinequivalent to the RLD. In each of the two cases, temporally resolved normalized intensity profiles similar to PK profiles were used to compare the products. One additional dimension was that the concentration of both active ingredients could be quantified at multiple depths in the skin as opposed to bulk one dimensional permeation data across the skin that is typically obtained using IVPT or PK studies. The authors concluded that, for BE metrics such as the AUC, using a 90% CI criteria, the methodology consistently concluded BE or lack thereof for each pairwise comparison completed in the study.

Kuzma et al.^75^ reported the use of coherent Raman scattering imaging (CRI) as a methodology to reduce sample-to-sample variability and standardize reproducible quantification of API within human skin tissue, which in turn enables reliable data collection across experiments. Because evaluation of BA or BE usually includes comparison of multiple formulations across multiple samples, which may be collected over days to weeks, standardization of data collection and reducing intra- and inter-individual variability are paramount to being able to use the data for comparing formulation, especially to support an evaluation of BE.

Iliopoulos et al.^76^ and Tu et al.^77^ reported the use of an SRS microscope for time resolved quantification of tazarotene and tretinoin in the skin from approved topical formulations. Although the design of the studies was similar to the previously cited work in that in each case an RLD was compared with an approved generic that is known to be BE and alternate lab-made formulations that are anticipated to be nonBE, use of the SRS compared with the confocal Raman resulted in shorter data acquisition times and therefore significantly higher temporal resolution for the skin intensity data. The SRS system also allowed for higher depth resolution. For both tazarotene, and tretinoin the results suggested that the methodology employed in the study is able to reproducibly collect temporally and spatially resolved skin concentration data for the active ingredients against the background of the skin and the other components in the formulations. The data were subsequently used to compare the BA of the active ingredient from the formulations and evaluate BE.

Link et al.^78^ utilized a gen2-SCA Skin Analyzer (RiverD International B.V., Rotterdam, Netherlands) to compare four formulations containing salicylic acid in 12 healthy human volunteers at two different timepoints. The authors concluded that the study successfully demonstrated BE for the products that were anticipated to be BE, and the control product (which was essentially a diluted form of the reference formulation) was found to be bioinequivalent.

Kim et al.^79^ utilized quantitative volumetric photoacoustic microscopy (PAM) for assessing vasoconstriction in the skin following topical application of corticosteroids. Historically, chromameters have been used as a surrogate measure of corticosteroid potency by quantification of the skin blanching effect of the corticosteroids. However, the current work illustrates how imaging-based tools can be used to directly visualize and quantify the vasoconstriction effect following application of corticosteroids; the results can be further used to compare formulations and assess the relative potency of corticosteroid formulations.

Analysis powered by trained convolutional neural networks has been used for coherent Raman and SRS data to precisely identify lipid-rich and lipid-poor regions in the skin, and label-free quantification of spatially and temporally resolved skin concentration data in the skin in each of the two regions of interest.^73^^,^^74^^,^^80^

In summary, the recent literature suggests that confocal Raman, SRS, and photoacoustic imaging-based tools are available for noninvasive quantification of drug concentration in the skin following topical application; however, further enhancements are needed for broad applicability of the tools across different active ingredients and dosage forms.

### 3.2 Drug Delivery to the Eye

For many complex ophthalmic products, as previously noted, establishing in vitro Q3 sameness is necessary but may not be sufficient to fully demonstrate BE, particularly when the generic product contains permissible differences in functional excipients (e.g., viscosity enhancers and preservatives) that can dynamically interact with formulation components and/or the ocular surface. Traditional PK studies often rely on sampling aqueous humor or analyzing systemic blood concentrations. However, systemic exposure following topical ocular administration is frequently too low to be quantified, and bulk tissue homogenization fails to capture the precise anatomical distribution of the drug across the eye’s multiple barrier layers. Furthermore, FDA analyses of aqueous-humor PK studies submitted for topical ophthalmic corticosteroid suspensions further showed that age and ethnicity can significantly affect aqueous-humor PK endpoints, underscoring the need for carefully designed PK studies and strengthening the case for increasingly relying on orthogonal, imaging-based evidence when establishing BE.^81^

In the preclinical development of generic ophthalmic formulations, accurately mapping the biodistribution of the active ingredient is helpful for confirming that the test and RLD deliver the drug to the target tissue at the equivalent rate and extent. Matrix-assisted laser desorption/ionization mass spectrometry imaging (MALDI-MSI) is increasingly used for preclinical ocular PK because it enables label-free, high-resolution mapping of drug distribution within intact ocular tissue sections.^82^ By utilizing MALDI-MSI in animal models, developers can visualize spatial ocular distribution after local administration. This can provide direct spatial evidence that the generic formulation achieves the same local concentration gradients in target tissues, such as the ciliary body or retina, supporting a conclusion of equivalent delivery within the preclinical context.

Furthermore, for long-acting injectable suspensions or implants, understanding the in vivo behavior of the drug depot is essential. Modalities such as X-ray, CT, and other non-invasive longitudinal approaches can track depot morphology and structural change over time in the same living animal. These imaging tools can potentially detect if a generic intravitreal suspension agglomerates differently than the reference or if an implant migrates or erodes at an accelerated or reduced rate, thereby informing formulation adjustments before advancing to costly clinical BE studies.

Beyond high-resolution spatial mapping, ocular fluorophotometry serves as a vital preclinical tool for the supportive characterization of the formulation’s delivery vehicle.^83^^,^^84^ By incorporating a fluorescent tracer (e.g., sodium fluorescein) into the generic formulation and RLD, developers can quantify the precorneal residence time and clearance kinetics in animal models. On a supportive basis, preclinical fluorophotometry allows for the head-to-head comparison of test and RLD residence and clearance profiles. A meaningful difference in clearance rate for the generic product suggests it does not fully match the relevant *in vitro* Q3 properties required for equivalent in vivo performance; such a discrepancy would likely lead to a failure in establishing BE. Using fluorophotometry early in the development cycle, researchers can optimize their products to ensure the generic formulation provides an identical temporal window for drug absorption as the RLD.

In human clinical trials, where invasive tissue sampling is ethically and practically precluded, establishing BE for locally acting ophthalmic products has traditionally relied on subjective CCEP BE studies (e.g., visual grading of inflammation via slit-lamp). These subjective assessments inherently introduce high variability, often necessitating large, expensive patient cohorts to achieve statistical significance. Specialized optical imaging techniques can potentially overcome this barrier by providing objective, non-invasive surrogate measures of both PK and PD, directly capturing drug behavior and kinetics in target compartments such as the tear film and the aqueous humor non-invasively.^85^ If a generic product possesses different rheological or mucoadhesive properties compared to the RLD, fluorophotometry may detect a divergence in the clearance profile. This can serve as a highly sensitive indicator of potential BE failure directly in humans.

In the preclinical and clinical *in vivo* settings, AI is transforming how we interpret tissue distribution and PK/PD imaging. Machine learning models excel at automating the segmentation of distinct ocular anatomical layers in AS-OCT or retinal OCT scans, rapidly calculating precise metrics such as corneal thickness (pachymetry) or tracking the erosion volume of an intravitreal implant over time.

## 4 Advances in Imaging Technologies for Characterization of Drug Delivery Devices

Many complex ophthalmic products rely not just on the formulation but on specialized delivery devices, such as multi-dose droppers or ophthalmic sprays. To establish BE, a generic product must demonstrate that its device delivers the formulation in a manner comparable to the RLD so that it does not impact BE. High-speed optical imaging and laser-based techniques are routinely deployed to evaluate the fluid dynamics and delivery characteristics of these complex devices.^86^ For ophthalmic sprays, high-speed photography combined with phase Doppler anemometry (PDA) or laser diffraction can be used to meticulously characterize critical parameters such as plume geometry, spray pattern, and droplet size distribution.^87^ These parameters are important because a generic spray with a narrower plume or a significantly different droplet size distribution may alter the deposition pattern on the ocular surface, potentially leading to localized toxicity or a failure to achieve therapeutic exposure across the cornea.

Similarly, for eyedroppers, high-resolution optical imaging can be utilized to dynamically measure drop volume, drop shape, and drop formation dynamics at the orifice. Because the human eye can only hold a limited volume of fluid before reflex tearing and spillage occur, variations in drop volume delivered by a generic device can drastically alter the actual dose retained in the cul-de-sac when dosing below its volume capacity.^16^^,^^18^ Furthermore, advanced OCT and high-speed videography can be employed *ex vivo* or *in vivo* to observe the physical interaction of the drop upon impact with the ocular surface..^88^^,^^89^ This includes visualizing the spreading kinetics and the instantaneous mixing of the formulation with simulated or human tear film.

For inhalation products, SEM coupled with AFM (SEM/AFM) was used to characterize neck gasket topography MDI valves after exposure to HFA 134a, HFA 227ea, HFA152a, and HFO 1234ze.^90^ The SEM/AFM imaging revealed the nitrile gasket had a rough scale-like surface structure, whereas a cyclic olefin copolymer (COC) valve had a much smoother surface. The increase in surface roughness in the nitrile valves was thought be attributable to increased surface area for greater interaction with gas molecules that likely contributed to higher propellant permeation rates. The COC-based valves minimized molecular diffusion and gas permeation by reducing effective contact area between gas molecules and the gasket. Thus, the SEM/AFM method was able to successfully support differences in leakage rates of these MDI propellants when using different valve materials and likely improve the quality of MDIs when transitioning from HFA propellants to NGPs.

## 5 Advances in Imaging Technologies to Support Development of in Silico Models

An area of interest for inhalation drug products is the use of imaging modalities to support the building and validation of in silico models. Work generated by Contract 75F40123C00201 with the University of Sydney developed high speed imaging (HSI) and optical coherence tomography (OCT) techniques to characterize powder flow behavior of inhalation powders within and emitted from DPIs.^91^^–^^94^ HSI was conducted to analyze particle size and velocities of inhalation powders on commercial products and within acrylic versions of common DPI designs (e.g., swirl, chamber, and capsule chamber). The developed OCT methodology was able to capture deposition thickness distribution and dynamic deposition behavior in a DPI device. Such techniques allow for analysis of formulation powder differences study of the impact of powder flow dynamics within and emitted from DPI devices. Further analysis is ongoing to tie powder flow behavior to aerodynamic performance. In addition, these key studies provide *in vitro* datasets that can help support the building and validation of in silico models by providing key insights to powder flow behavior within and emitted from various DPI device designs. Lastly, FDA-funded Grant 1U01FD007987-01 with Fluidda Inc., was awarded to develop and validate *in vivo* lung deposition techniques with nuclear medicine imaging methods. Such work is ongoing but is projected to provide direct lung deposition data from in vivo imaging that can be used to validate in silico models and provide additional datasets capable of building *in vitro-in vivo* relationships. The importance in building links between in vitro imaging and in silico models is likely to support flexibility in critical assessments of inhalation products in the future as these in silico models are likely to be more prevalent. Model-integrated approaches have the potential to streamline generic drug development by providing more scientific and biorelevant parameters for evaluating product performance, by potentially allowing pathways in which *in vitro* and in silico models could serve as surrogates to *in vivo* studies, and by supporting the model master file concept.^95^^,^^96^

## 6 Conclusion

GDUFA-supported research to advance imaging technologies for generic drugs has produced outcomes of substantial regulatory significance for multiple types of complex drug products. Non-destructive Q3 characterization is now possible for dosage form microstructures that were previously inaccessible. Quantitative structure-performance correlations have connected structural attributes to drug release kinetics in a mechanistically predictive manner. Imaging-based methods have enabled the direct measurement of drug availability at local sites of action. AI/ML has provided the analytical infrastructure needed to extract meaningful insights from high order imaging data. And, additionally, mechanistic computational models informed by imaging-derived structural data have generated totality-of-evidence arguments grounded in structure-performance relationships.

It is notable that the approval of the first generic mometasone furoate metered nasal suspension spray relied on MDRS-derived drug particle size distribution data to characterize the active ingredient-specific PSD in the presence of suspended excipients, supporting a structural equivalence determination that eliminated the need for a comparative clinical endpoint BE study.^33^ That approval established a scientific precedent and a PSG framework subsequently extended to additional nasal suspension products.^35^^,^^36^ The approval of the first U.S. generic cyclosporine ophthalmic emulsion 0.05% in February 2022, was built upon more than a decade of GDUFA-funded physicochemical and imaging-based characterization research and illustrates the translational power of a sustained science and research program for generic drugs.^97^

Being able to visualize and quantify where the active ingredient and/or excipient resides within a multiphasic formulation, how the drug diffuses and is released from the dosage form, the delivery and disposition of the drug, and performance of the dosage form can be a powerful tool towards the development of dermal drug products. The FDA’s generic drug research program and the collaborations between FDA scientists, academic research partners, and the generic drug industry have successfully advanced multiple imaging technologies to support demonstrations of BE. This includes research initiatives that have developed new tools for *in situ* Q3 characterizations, structure-performance correlations, direct measurement of drug availability at the site of action, AI-enabled imaging data analysis, and mechanistic computational modeling. These advances collectively provide powerful new technologies to assist the development and the regulatory assessment of complex generic drug products. However, to realize their full potential, these technologies ideally need to be incorporated into commercially available instruments with capabilities that are optimized for the study designs needed in pharmaceutical product research and development and for regulatory decision-making. These capabilities include substantially reduced acquisition times, better spatial resolution, automated high-throughput sample analysis, integrated and validated data processing, and user-friendly software interfaces. Such instruments would be able to support demonstrations of BE for numerous complex generic products that can expand patient access to safe, effective, and high-quality generic alternatives across topical, ophthalmic, nasal, inhalation, complex injectable, and long-acting injectable products.

## Data Availability

Data sharing is not applicable to this article, as no new data were created or analyzed.
